# Impact of Oral Side Effects from Conditioning Therapy Before Hematopoietic Stem Cell Transplantation: Protocol for a Multicenter Study

**DOI:** 10.2196/resprot.8982

**Published:** 2018-04-23

**Authors:** Michael T Brennan, Bengt Hasséus, Allan J Hovan, Judith E Raber-Durlacher, Nicole MA Blijlevens, Marie-Charlotte Huysmans, Karin Garming Legert, Jan-Erik Johansson, Charity G Moore, Inger von Bültzingslöwen

**Affiliations:** ^1^ Department of Oral Medicine Carolinas Healthcare System Charlotte, NC United States; ^2^ Department of Oral Medicine and Pathology University of Gothenburg Gothenburg Sweden; ^3^ BC Cancer Vancouver, BC Canada; ^4^ Department of Oral Medicine Academic Centre for Dentistry Amsterdam Netherlands; ^5^ Department of Oral and Maxillofacial Surgery Academic Medical Center Amsterdam Netherlands; ^6^ Radboud University Medical Center Nijmegen Netherlands; ^7^ Karolinska Institute Huddinge Sweden; ^8^ Department of Hematology and Coagulation Sahlgrenska University Hospital Gothenburg Sweden; ^9^ Department of Physical Therapy University of Pittsburgh Pittsburgh, PA United States; ^10^ Institute of Odontology Sahlgrenska Academy Gothenburg Sweden

**Keywords:** mucositis, hematopoietic stem cell transplantation, chemotherapy, xerostomia, cohort studies, graft vs host disease, dental disease, multicenter study, oral cavity, costs and cost analysis

## Abstract

**Background:**

The oral cavity is a common site of complications related to the cytotoxic effect of high-dose chemotherapy and radiation therapy. Considering our limited understanding of the burden of illness in the oral cavity from various cytotoxic therapies, it is difficult to produce evidence-based, preventive and management protocols. A prospective multicenter study is necessary to collect data on the burden of illness from various cytotoxic regimens.

**Objective:**

The objectives of this prospective international observational multicenter study in hematopoietic stem cell transplant (HSCT) patients are to establish the nature, incidence and temporal relationship of oral complications related to conditioning regimens (chemotherapy with or without total body irradiation), stem cell transplantation and the immunologic reactions (mainly graft-vs-host-disease) that may follow, and to determine what subjective and objective oral complications related to treatment can predict negative clinical and economic outcomes and reduced quality of life.

**Methods:**

Adult patients at six study sites receiving full intensity conditioning, reduced intensity conditioning or nonmyeloablative conditioning, followed by autologous or allogeneic hematopoietic stem cell infusion, are included. A pre-treatment assessment includes medical conditions, planned chemo- and radiation therapy regimen, medications, allergies, social history, patient report of oral problems, dental history, subjective oral complaints, objective measures of oral conditions, current laboratory values, dental treatment recommended and untreated dental disease. Starting 1-3 days after hematopoietic stem cell infusion, a bedside assessment is completed 3 days per week until resolution of neutropenia. A patient questionnaire is also completed during hospitalization. Beyond this time, patients with continued oral mucositis or other oral problems are followed 1 day per week in an inpatient or outpatient setting. Additional visits for urgent care for acute oral problems after hospitalization are documented. Autologous transplant patients are being followed up at 100 days (SD 30 days) and at 1 year (SD 30 days) post-transplantation to identify any long-term side effects. Patients treated with allogeneic transplantation are being followed at 100 days (SD 30 days), 6 months (SD 30 days), and 12 months (SD 30 days). The follow-up assessments include cancer response to therapy, current medical conditions, medications, subjective and objective oral findings, quality of life measures and laboratory values. The targeted enrollment is 254 patients who have received HSCT.

**Results:**

A total of 260 participants have been enrolled, with 233 (91%) who have received HSCT. We anticipate enrollment of 20-30 additional participants to obtain the sample size of 254 enrolled participants who have received HSCT.

**Conclusions:**

The results of the ongoing prospective study will provide a unique dataset to understand the impact of oral complications on patients undergoing HSCT and provide needed evidence for guidelines regarding the management of this patient cohort.

## Introduction

The annual incidence of cancer is 11 million cases worldwide [1]. Early cancer detection and advances in cancer therapies have provided important management advances to improve survival, and quality of life (QOL) in later years. Common cancer treatment strategies include surgical resection, chemotherapy (CT), radiotherapy (RT), and hematopoietic stem cell transplantations (HSCT). The goal of such treatments is to eliminate all cancer cells. However, side effects from these therapies can limit the effectiveness of treatment and have a marked impact on the patient’s QOL. The oral cavity is a common site of complications related to cytotoxic therapies. The Surgeon General’s report on Oral Health in America estimates that more than 400,000 patients in the US undergoing cancer treatment will develop oral complications annually [2]. The report calls for more science and the effective translation of science to improve oral health and establish clinical practice guidelines based on a higher quality science base. Recommendations are needed to effectively transfer research findings in the field of oncology, including oral complications seen with cancer therapies, and oral management prior to the start of cancer therapy, to public and health professionals. Well developed, evidence-based management recommendations have the potential to enhance the appropriateness of clinical practice, improve the quality of oral health care, lead to better patient outcomes, and identify areas of further research needs. To effectively change perceptions of the burden of illness of oral complications from cancer therapies, a complete understanding of the impact of these complications is vital. Underestimating the impact of oral complications may result in avoidance or delay in appropriate care for cancer patients.

Numerous preventive care protocols have been proposed to minimize oral complications from cancer therapies. Unfortunately, these protocols are rarely evidence-based and often rely on “expert opinion” or anecdotes. The lack of well-controlled, prospective studies is the primary reason for the limitation in preventive and management protocols. The Institute of Medicine (IOM) report determined that insufficient systematic research is available to assess the prevention and management of the oral problems associated with head and neck cancer, leukemia, and lymphoma [3].

Considering our limited understanding of the burden of illness in the oral cavity from various cancer therapies, it is difficult to produce evidence-based, preventive and management protocols. Therefore, a prospective multicenter study is necessary to collect data on the burden of illness from various cancer regimens.

The literature reports a wide range of oral complications with varying incidences. Oral mucositis, or inflammation of the mucosal surfaces, sometimes also called mucosal barrier injury (MBI), is a major dose-limiting side effect of chemo- and radiation therapy, specifically conditioning therapy before HSCT. Severe mucositis has been associated with pain, infection, poor nutrition, increased hospitalization and a major impact on QOL and economic outcomes. Other reported complications are bleeding, dysphagia (difficulty swallowing), dysgeusia (altered sensation of taste), infection (bacterial, viral, and fungal), pain, trismus, medication-related osteonecrosis of the jaw, osteoradionecrosis, xerostomia/salivary gland dysfunction, caries, periodontal disease, and graft-vs-host-disease (GVHD) [4]. Recent systematic reviews of these oral complications have confirmed the limitations in knowledge of the incidence and severity of the various additive oral complications [5]. Additionally, based on these systematic reviews, prevention and management protocols are very limited in quality and design such that it is difficult to produce evidence-based protocols leading to a significant gap in treatment protocols for this patient population. Furthermore, how these common oral complications impact clinical and economic outcomes and affect (QOL) is poorly understood [6]. The goal of the present study is to bridge these research gaps by way of a well-designed, prospective, multicenter observational study.

The objectives of this prospective international observational multicenter study in HSCT patients is to determine the relevant factors that may predict negative clinical and economic outcomes through the following methods and approaches. We will establish the nature, incidence and temporal relationship of oral complications related to conditioning regimens (chemotherapy with or without total body irradiation, HSCT and the immunologic reactions (mainly chronic GVHD) that may follow, and to determine what subjective and objective oral complications related to treatment can predict negative clinical and economic outcomes and reduced QOL.

The purpose of this manuscript is to describe the study protocol for this important ongoing multi-center study and to report the current sites and enrollment.

## Methods

### Study Design

The present study aims to address research gaps regarding the impact of oral complications in HSCT patients by way of a well-designed prospective, longitudinal, international, observational, multicenter cohort study of patients receiving conditioning regimens followed by HSCT (autologous or allogeneic). Data collected from this study will allow a comprehensive understanding of the burden of illness of oral complications related to type of conditioning therapy and provide a clearer understanding of prevention and management protocols for oral complications in HSCT patients.

### Outcome Measures

To address the primary aim of determining the incidence, severity and temporal relationship of oral complications related to type of conditioning regimen, we register demographics (age, sex), diagnosis, cytotoxic therapy and assess the following outcomes (details are provided in Multimedia Appendix 1):

- Subjective oral complications: Oral pain, xerostomia (dry mouth), dysgeusia (taste changes) and dysphagia (swallowing difficulties);
- Objective oral complications: Oral mucositis, hyposalivation, oral infections (viral, fungal, bacterial), submucosal hemorrhage, dental and periodontal diseases and complications, osteonecrosis, and GVHD.

To address the secondary aims of determining what factors can predict negative clinical and economic outcomes and reduced QOL, we assess the following:

- Antimicrobial prophylaxis or Keratinocyte Growth Factor medication and ongoing immunosuppression for GVHD
- Institutional standard of care preventative and management protocols
- Nausea or vomiting, diarrhea, fever, weight and blood values
- Additional hospital visits, prolonged hospital stays, systemic infection, increased medication/treatment (eg antibiotics, opioids), poor nutrition or parenteral nutrition and death.
- Subjective measures of oral pain, xerostomia, dysgeusia and dysphagia, and a feeling of well-being. To minimize the length of time needed to participate in the present study, generic oral health-related quality of life measures (eg, OHIP-14) were not used and instead QOL questions were focused on subjective areas related to oral complications from cancer therapy.
- Genomic factors (Multimedia Appendix 2).

### Study Organization

Subjects are enrolled at six clinical study sites: Sahlgrenska University Hospital, Gothenburg, Sweden; Karolinska University Hospital Huddinge, Stockholm, Sweden; Carolinas Medical Center, Charlotte, NC, USA; BC Cancer, Vancouver, BC, Canada; Academic Medical Center, Amsterdam, The Netherlands; Radboud University Medical Center, Nijmegen, The Netherlands. Study personnel at each site include the site principal investigator, study coordinator(s), and clinical examiner(s). The Data Coordinating Center for the study is located at the Carolinas HealthCare Systems, Charlotte, NC, USA.

### Safety of Human Subjects and Data Integrity

Approval from the research ethics board at each clinical study site was obtained prior to enrollment. Sites were approved to start enrollment after a site visit was completed by the study principal investigators (IVB, MTB) to ensure appropriate infrastructure, patient population, research staffing, calibration of research personnel of the study design, data management and study outcomes. Informed consent is obtained from each study participant prior to inclusionStudy data is entered into MedView, which is a computer program that is based on formalized input and registration of all clinical information. MedView provides a suite of tools for formalizing, gathering, and analyzing data. MedView is aimed at clinical research and is well suited for multicenter studies [7]. MedView program is accessible on the Internet. Each participating center is provided with a unique username and password. Data sent over the internet are encrypted. No personal identifiers are included. Study data is loaded into a secure database at University of Gothenburg, Sweden. Staff from the Data Coordinating Center monitors validation of the study data to identify missing data or forms or incorrect registrations and communicates this information with each enrollment site to resolve any problems.

### Training and Calibration

All study personnel receive training on the parameters needed to conduct this study which includes training on clinical assessments; training on completing study forms, data entry and all non-clinical procedures. In addition, annual calibration on objective clinical measures is conducted for all clinical examiners.

### Subject Selection Criteria

#### Inclusion Criteria

Adult patients receiving full intensity conditioning, reduced intensity conditioning or nonmyeloablative conditioning, followed by hematopoietic stem cell infusion (autologous or allogeneic) are eligible for inclusion. For diagnoses, see Textbox 1.

#### Exclusion Criteria

Patients unable to give consent are not eligible for inclusion.

**Patients with the following diagnoses to go through hematopoietic stem cell transplantations (HSCT) are eligible for inclusion.**

**For allogeneic transplant:**

Acute leukemia

Myelodysplastic syndrome

Aplastic anemia

Lymphoma including chronic lymphocytic leukemia

Chronic myeloid leukemia

Myelofibrosis

Other conditions being managed with HSCT

**For autologous transplant:**

Multiple myeloma

Lymphoma including chronic lymphocytic leukemia

Testicular cancer

Autoimmune diseases (scleroderma; systemic sclerosis)

Other conditions being managed with HSCT

**Figure 1:**
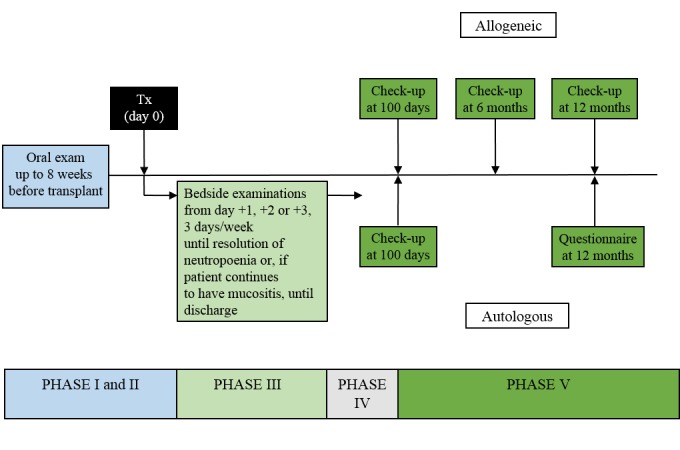
Study flow diagram.

#### Study Assessments

Patients who meet the enrollment criteria are approached and consent is reviewed and obtained. A pretreatment assessment (Phase I and II) is completed to include the following: medical conditions, planned chemo- and radiation conditioning therapy regimen, medications, allergies, social history, patient report of oral problems, dental history, subjective oral complaints, objective measures of oral conditions, current laboratory values, dental treatment recommended and untreated dental disease. This pretreatment assessment will occur up to 8 weeks prior to stem cell transplantation (Figure 1).

### Study Flow Diagram

A bedside assessment (Phase III) will be completed 3 days per week (Monday, Wednesday, Friday) starting day +1, +2 or +3 after transplantation (day 0) depending on which day of the week the transplantation occurs, until resolution of neutropenia (ie, absolute granulocyte count > 0.5 x 10^9^/l). For patients with continued mucositis or other oral problems requiring hospitalization after resolution of neutropenia, an oral examination will be completed for up to 6 weeks duration. A patient questionnaire will be completed for each study visit to the patient (Monday, Wednesday, Friday) during the patient hospitalization. Beyond this time, patients with continued oral mucositis or other oral problems will be followed 1 day per week (Phase IV) in an inpatient or outpatient setting.

Additional visits for urgent care for acute oral problems will be documented regarding the nature of the oral problem and treatment provided (Phase IV). This will be documented for up to 6 months for the autologous stem cell transplantation patients and 12 months for the allogeneic transplant patients.

For long-term follow-up, patients with autologous transplantation will be followed-up as part of standard of care visits. The first follow-up visit (Phase V) in an outpatient setting will occur 100 days (SD 30 days). The patients will also complete the patient questionnaire at 100 days (SD 30 days). These patients will also receive a questionnaire (Phase VI) by mail at 1 year (SD 30 days) posttransplantation to identify any long-term side effects **.** Patients with allogeneic transplantation will also be followed up as part of standard of care visits (Phase V) in an outpatient setting at 100 days (SD 30 days), as well as after 6 months (SD 30 days), and 12 months (SD 30 days), at which time the patient questionnaire is also completed. The follow-up assessments will include cancer response to therapy, current medical conditions, medications, subjective and objective oral findings, QOL measures and laboratory values. For the study outline of the standardized examination process, see Figure 1. If a patient is deceased, it will be noted (Phase VII).

### Statistical Considerations

Analyses for establishing the nature of oral complications will be primarily descriptive in nature using percentages and rates with corresponding confidence intervals. Analyses to determine what oral complications related to treatment can predict negative outcomes will use inferential statistics comparing those with oral complications to those without. For each outcome, univariate analysis will use Student’s *t*-test (or Wilcoxon Rank Sum) for continuous measures and chi-square or Fisher’s exact tests for dichotomous variables with a critical value of 0.05. Additional potential risk factors that are thought to have prognostic value will first be analyzed by univariate analysis and appropriate variables (*P*<0.1) will be considered as confounders in multivariable modeling (either linear or logistic regression depending on the outcome distribution).

**Table 1:** Expected incidence of oral complications after HSCT and estimated sample size required to reach a statistical significance level of 95%. GVHD: graft-vs-host-disease.

Oral complication	Estimate source	Expected incidence (%)	Sample size	Precision (%)
Mucositis	Preliminary study	65	237	+/– 6
			133	+/– 8
			83	+/– 10
Xerostomia	[8]	40	244	+/– 6
			140	+/– 8
			86	+/– 10
Oral pain	[9]	45	251	+/– 6
			148	+/– 8
			91	+/– 10
Dysphagia	Preliminary literature review	54	252	+/– 6
			139	+/– 8
			93	+/– 10
Chronic GVHD	[10]	7-54	62-252	+/– 6
Dysgeusia	Preliminary study	38	246	+/– 6
			133	+/– 8
			88	+/– 10
Oral viral	[11]	43	254	+/– 6
			148	+/– 8
			93	+/– 10
Oral fungal	[12]	38	252	+/– 6
			142	+/– 8
			91	+/– 10

Adjusted differences in means or adjusted odds ratios and corresponding confidence intervals will be calculated to represent measures of association. Analyses will be performed with the SAS Enterprise Guide version 6.1 (SAS Institute Inc, Cary, North Carolina, USA).

### Sample Size

The primary aim of the study is to establish the nature, incidence and temporal relationship of oral complications related to conditioning regimen. The source of the expected incidences is included in Table 1. These samples sizes were determined with alpha of .05 and differing levels of acceptable absolute precision. The range of sample size estimates for the various oral complications ranged from 62-254 depending on the incidence estimate and precision level. Using the most conservative estimate, a total of 254 patients will need to be assessed for oral complications during stem cell transplantation to obtain 6% precision for the main oral complications listed in Table 1. Thus, to account for participants that do not make it to stem cell transplant (approximately 10%) and to ensure sufficient patients are assessed to allow for differences in enrollment sites, we will enroll up to 320 patients. The present study is a prospective, observational registry, with no interventional component. We anticipate that there will be differences in approach to oral prevention and management protocols per treatment center. This will provide an opportunity to describe the impact of different management regimens on Ora-Stem outcomes. The study was not originally powered for these differences; thus, this data may be more preliminary in nature, but still provide a robust dataset to explore differences in enrollment sites.

## Results

To date, 91% of a targeted number of participants have been enrolled with 233 participants receiving an HSCT. We anticipate enrollment of at least 20-30 additional participants to ensure 254 enrolled patients receive a HSCT, which will be completed by June 2018.

A preliminary analysis completed October 30, 2017 of 222 enrolled participants demonstrated the most common medical diagnoses managed with HSCT included: multiple myeloma=34%; acute myelogenous leukemia=22%; lymphoma=15%, and acute lymphocytic leukemia=6% with 53% managed by allogeneic transplantation. Preliminary assessment of oral complications demonstrated approximately 32% of all participants experienced a grade 2 or higher mucositis during hospitalization and at least 39% of allogeneic HSCT patients have developed oral GVHD (this is an underestimation as not all patients have completed a follow-up visit).

## Discussion

### Rationale

Numerous oral complications have been associated with cytotoxic therapies. To establish recommendations for pre-, interim-, and postcancer therapy management of oral problems in patients receiving high dose conditioning regimen and HSCT (autologous or allogeneic), an understanding of the scope of oral complications from HSCT must be established and be related to time after treatment and treatment regimen. The lack of clarity in this field is reflected in a lack of comprehensive and effective oral management regimens in the clinical arena. With a deeper understanding of oral complications, oral care regimens to minimize such complications can be appropriately formulated and evaluated. There is thus a pressing need to establish the nature, incidence and temporal relationship of oral complications related to conditioning therapies, as well as other types of chemo- and radiation therapies.

### Limitations

To optimize the data quality and generalizability of the present study, a prospective, multicenter design was planned. Due to limitations in research infrastructure, it was not possible to consecutively enroll patients at every site. Although patients are not consecutively enrolled due to logistical limitations of enrollment of all possible patients; deliberate efforts to ensure high data quality from patients who were enrolled will allow for a robust dataset. We have also tracked and will report on all patients that could have been enrolled and the reason they were not enrolled. Additionally, the current study is observational in nature only and thus relies on institutional standard of care preventative and management protocols. This will allow for comparisons of the different treatment protocols between the enrollment sites. The design of this study is purposefully not an interventional study to allow for this comparison of standard of care protocols, thus definitive data on effectiveness of protocols will not be possible with this study design but will be instrumental in the design of future interventional studies.

### Conclusions

The results of the ongoing prospective study will provide a unique dataset to understand the impact of oral complications on patients undergoing HSCT and provide needed information with forming more evidence-based guidelines regarding the management of this patient cohort.

## Appendix

Multimedia Appendix 1 Outcomes: Oral findings (complications) to be included in the study.

Multimedia Appendix 2 Saliva samples for functional genomic studies.

## Abbreviations

HSCT: hematopoietic stem cell transplant
GVHD: graft-vs-host disease
QOL: quality of life
