# Supplementary material for: Impact of Oral Side Effects from Conditioning Therapy Before Hematopoietic Stem Cell Transplantation: Protocol for a Multicenter Study
Source: JMIR Res Protoc. 2018 Apr 23;7(4):e103. doi: 10.2196/resprot.8982 (PMC5938569; doi:10.2196/resprot.8982)
Supplement: Multimedia Appendix 1 [file resprot_v7i4e103_app1.pdf]

Subjective Oral Findings/Complications

Finding/Complication	Method of data collection	Instrument	Timing (Phase)
Oral pain (severity and location)	Validated scales	NCI CTCAE v.3 [13]<br>WHO pain ladder [14]	III
Xerostomia (dryness vs viscosity)	Scale	NCI CTCAE v.3 [15]<br>VAS scale	I, III, V
Taste changes (Dysgeusia- abnormal vs absent)	Scale	Epstein scales [16]	III
Dysphagia (difficulty to eat, speak and swallow)	Scale	NCI CTCAE v.3 [15]	III

Objective Oral Findings/Complications

**Finding/Complication**	**Method of data collection**	**Instrument**	**Timing (Phase)**
Oral mucositis	Scales	WHO [17]- Oral Mucositis Assessment Scale (OMAS) [18]	II, III, IV, V, III, IV
Oral infections: viral, fungal or bacterial	Clinical judgement-culture	Clinical examination and description	II, III, IV, V
Submucosal hemorrhages	Clinical judgement	Clinical examination and description	II, III, IV, V
Existing dental and periodontal disease	Clinical judgement/diagnosis	Clinical and radiographic examination of: number of remaining teeth/implants, caries, root canal treated teeth, chronic apical periodontitis, partially erupted wisdom teeth, plaque, calculus, periodontitis (pocket depth and bleeding on probing)	I, II
Dental and periodontal complications during cancer therapy	Clinical judgement/diagnosis	Clinical and radiographic examination of: pulpitis, abscesses, pericoronitis, gingival bleeding	III, IV, V
Osteonecrosis	Clinical judgement/diagnosis	Clinical and radiographic examination	V
Oral GVHD	Clinical judgement/diagnosis	Clinical examination and description	III, IV, V
Stimulated salivary flow (paraffin/gum base chewing)	Salivary probe	5 minutes (mL/minutes)	I, V
Others	Photo documentation	Camera	I-V if indicated

Signs to predict oral complications

**Finding/Complication**	**Method of data collection**	**Instrument**	**Timing (Phase)**
Salivary sample (2 mL from stimulated whole saliva) for the genomic studies	Salivary probe	Genomic expression analysis	I

Confounding variables

**Finding/Complication**	**Method of data collection**	**Instrument**	**Timing (Phase)**
Demographics/social history	Patient records, interviewing	Age (year), sex, tobacco, alcohol	I
Diagnosis treated with HSCT	Patient records	Diagnosis name	I
Cytotoxic therapy	Patient records	Previous chemotherapy, radiotherapy and investigational therapies; conditioning regimen for current HSCT	I, II
Antimicrobial prophylaxis	Patient records	Type, dose, duration	II, III
Keratinocyte growth factor medication, Cryotherapy	Patient records	Type, dose, duration	III
Immunosuppression for chronic GVHD		Type	II
Recommendations for local special oral care programs and individualized recommendations		Type, dose, duration	III

Nausea		NCI-CTCAE v.3 [13]	III
Vomiting		NCI CTCAE v.3 [13]	III
Diarrhea		Number of episodes during last 24 hours (OMDQ questionnaire) [19]	III, V
Fever		Celsius	II, III, IV, V
Weight		Kg	II, III
Blood values		White blood cell ($x10^9$/l), Platelets ($x10^9$/l)	II, III, IV, V

General Clinical Outcomes resulting from Oral Findings/Complications

**Finding/Complication**	**Method of data collection**	**Instrument**	**Timing (Phase)**
Systemic infection	Patient records	Type of infection, symptoms, duration, CRP	II, III, IV, V
Additional antibiotic therapy	Patient records	Type, dose, duration	III, IV, V
Narcotic analgesics	Patient records	Oral or IV, Type, dose, duration	III, IV, V
Other additional medication	Patient records	Type, dose, duration	III, IV, V
Nutrition	Patient records	Total parenteral nutrition; enteral nutrition (feeding tube); solid, liquid, enteral vs parenteral	III, IV
Weight loss	Patient records	Kg	III, IV, V
Survival	Patient records	Y/N, days	III, IV, V

Economic Outcomes resulting from Oral Findings/Complications

**Finding/Com-plication**	**Method of data collection**	**Instrument**	**Timing (Phase)**
Days of hospital-ization	Patient records	Days	III, IV
Emergency de-partment or ad-ditional hospital visits	Patient records	Number of visits, location of visits, timing (days fol-lowing transplantation)	III, IV
Emergency dental consultations	Clinical examina-tions or patient his-tory	Number of visits, location of visits, timing (days fol-lowing transplantation)	III, IV, V

Quality of Life (QOL) variables potentially affected by oral complications

**Finding/Complication**	**Method of data collection**	**Instrument**	**Timing (Phase)**
QOL issues related to symptoms from the oral cavity	Questionnaire	Oral mucositis daily questionnaire (OMDQ) [19]	III, V
Global questions on effects of oral cavity on general well-being	Questionnaire	Study specific Ora-Stem questions	III, V

Dental disease measures

Periodontology measures

- *Oral hygiene, measured in Phase II and III*

Baseline oral hygiene will be measured by the number of tooth surfaces with plaque present.

Visible plaque will be registered for each tooth:

  - No visible plaque
  - 1-20% of teeth with plaque will be graded as good oral hygiene
  - 21-50% of teeth with plaque is considered intermediate hygiene
  - >50% of the teeth with plaque is considered poor oral hygiene
- *Calculus, measured in Phase II*

Calculus is essential to measure since it facilitates the accumulation of plaque. It will be measured as:

- Supragingival calculus (yes/no) on any surface of the tooth. Registered tooth-by-tooth.
- Subgingival calculus (yes/no) on any surface of the tooth. Registered tooth-by-tooth.

The percentage of teeth with the presence of calculus will be reported.

- *Pocket depth, measured in Phase II (if possible)*

Full periodontal pocket depth index will be registered on four surfaces on each tooth at the dental clinic. The number of teeth with at least one pocket >5 mm (deep pockets) will be registered.

- *Bleeding on Probing (BoP), measured in Phase II (if possible)*

Bleeding will be noted (Y/N) with each tooth.

The percentage of teeth with the presence of bleeding on probing will be reported.

Saliva samples to measure salivation

The amount of stimulated whole saliva will be measured after 5 minutes of chewing paraffin/ tasteless gum base. To be collected at Phase I and at Phase V/100 days in both autologous and allogeneic transplant patients, in allogeneic transplant patients also at Phase V/1 year.

Xerostomia (subjective feeling of dry mouth) is evaluated in all phases. A question about xerostomia is also included in the 1 year-questionnaire to autologous transplant patients (Phase VI).
