# Supplementary material for: Impact of Oral Side Effects from Conditioning Therapy Before Hematopoietic Stem Cell Transplantation: Protocol for a Multicenter Study
Source: JMIR Res Protoc. 2018 Apr 23;7(4):e103. doi: 10.2196/resprot.8982 (PMC5938569; doi:10.2196/resprot.8982)
Supplement: Multimedia Appendix 2 [file resprot_v7i4e103_app2.pdf]

Saliva DNA samples will be obtained from patients prior to a conditioning regimen for autologous or allogeneic stem cell transplantation. These patients will be followed prospectively to document the incidence and severity of mucositis. We plan to use genomic approaches to determine candidate genes that will demonstrate an increased risk for the development of severe mucositis (WHO grade 3 or 4) and determine the genes' function in developing OM. Genomic approaches may include the use of sequence analysis methods (eg, SNP Arrays, Exome sequencing or other Next Generation Sequencing methods) for the determination of mutations (SNPs, indels [insertions/deletions], copy number variations [CNVs]), or the use of DNA methylation analytical methods (eg, Infinium HumanMethylation450 Illumina BeadChip). This will provide preliminary data to achieve a power of 80%, at least 2000 samples are required to identify mutations associated with susceptibility to OM considering an effect size between 1.3 and 1.6, which will require a substantial multicenter effort.

We may also generate DNA libraries which will guide future functional genomic studies.

A saliva sample will be obtained prior to the start of conditioning therapy and sent to Carolinas Medical Center. To keep collection of DNA as simple as possible, we will use the Oragene DNA system (DNA Genotek Inc. Ottawa, Ontario, Canada). Patients will rinse their mouth with water to clear any food debris before spitting 2 ml of saliva into the Oragene container. Once the container is closed, the contained reagents release the DNA from buccal epithelial cells in the saliva and stabilize it for long term storage. Samples can be kept at room temperature for several years without deterioration. The container is designed, approved, and comes with packaging for mailing. The median yield from a 2 ml saliva sample is 110 µg of DNA. This is higher than for most other oral epithelial cell sampling systems and more than adequate for any future genetic epidemiological studies that we may wish to pursue.

Following collection, the sample could be stored locally at room temperature before mailing

to the Carolinas Medical Center for long term storage at -20ºC. Samples stored at -20ºC can be kept almost indefinitely before further processing and analysis. The sample would be linked to data from the present study such as age, sex, medical conditions, conditioning therapy and oral complications. The sample will be coded by the enrollment site/patient sequential number (eg, A/053). None of the data collected with the questionnaires will contain personal health identifiers, therefore only researchers at the enrollment site will have a key to determine donor identity. This key will not be provided to other researchers. The samples will be stored for possible future genetic studies that are outside the remit of the current grant proposal. We anticipate seeking separate funding and ethical approval in the future for additional analysis of this material. Appropriate consent will be obtained for genomics studies based on our saliva DNA sample repository.
